# Exploring the relationship between immune heterogeneity characteristic genes of rheumatoid arthritis and acute myeloid leukemia

**DOI:** 10.1007/s12672-023-00852-7

**Published:** 2024-01-02

**Authors:** Chengzhi Jiang, Wenjuan Jiang, Pengtao Liu, Wenxue Sun, Wenjie Teng

**Affiliations:** 1 School of Public Health, Shandong Second Medical University, Weifang, Shandong 261053 People’s Republic of China; 2 Qingdao Municipal Hospital (Group), Qingdao, Shandong 266000 People’s Republic of China; 3 Computer Teaching and Research Office, Shandong Second Medical University, Weifang, Shandong 261053 People’s Republic of China

**Keywords:** Biomarkers, Acute myeloid leukemia, Machine learning, Therapeutic target

## Abstract

**Background:**

People with autoimmune diseases are prone to cancer, and there is a close relationship between rheumatoid arthritis (RA) and acute myeloid leukemia (AML). The bone marrow (BM) is affected throughout the course of RA, with a variety of hematologic involvement. Hopes are pinned on rheumatoid arthritis research to obtain BM biomarkers for AML.

**Methods:**

Synovial transcriptome sequencing data for RA and osteoarthritis (OA), and single-cell sequencing data for RA and controls were obtained from the GEO database.Bone marrow sequencing data for AML patients and normal subjects were obtained from the UCSC Xena database. The final immune heterogeneity characteristics of RA were determined through ssGSEA analysis, gene differential expression analysis, fuzzy c-means clustering algorithm, and XGboost algorithm. Random Ferns classifiers (RFs) are used to identify new bone marrow markers for AML.

**Results:**

SELL, PTPRC, IL7R, CCR7, and KLRB1 were able to distinguish leukemia cells from normal cells well, with AUC values higher than 0.970.

**Conclusion:**

Genes characterizing the immune heterogeneity of RA are associated with AML, and KLRBA may be a potential target for AML treatment.

## Introduction

A growing number of studies have highlighted a significant correlation between autoimmune diseases and malignancies. Specifically, cohort studies have demonstrated an elevated risk of leukemia in individuals with rheumatoid arthritis (RA), and an alarming surge of approximately 52% in acute myeloid leukemia (AML) incidence over the last decade (2009–2018) [1]. Importantly, both RA and AML involve profound alterations in the bone marrow microenvironment. In the context of RA, this autoimmune disease is characterized by the aggregation and infiltration of immune cells within the synovium prior to the manifestation of inflammation. Conversely, osteoarthritis (OA) is characterized by sterile inflammation without an immune response. Therefore, the synovium emerges as a pivotal site harboring potential immune heterogeneity differences between RA and OA. Although we have identified immunological heterogeneity features in RA, the expression and molecular mechanisms of these features in the context of AML remain unexplored.

In this study, we aimed to obtain relevant biomarkers by studying the immune heterogeneity of RA, to explore the relationship between these biomarkers and AML, to analyze whether they have the potential to be used as diagnostic biomarkers and therapeutic targets for AML, and to compare their diagnostic capabilities with those of the biomarkers that have been previously identified, such as CD13, CD33, CD14, and others [2–7]. To ensure the rigor and accuracy of the study, we adapted each machine learning model and mined a large amount of data in the study.

## Materials and methods

### Data acquisition

RNA sequencing datasets GSE36700 and GSE55584 for RA and OA were obtained from the GEO database (http://www.ncbi/nlm.nih.gov) with sample sizes of 25 and 16, respectively.The AML dataset GSE6891 (n = 536) with different cytological genetic risk profiles and CEBPA mutation types was also obtained from the GEO database. Mouse bone marrow single cell sequencing data GSM6893371 and GSM6893373 were also obtained from the GEO database. RNA sequencing dataset GDC TCGA Acute Myeloid Leukemia for AML was obtained from UCSC Xena (https://xenabrowser.net/datapages/) with a sample size of 151.RNA sequencing dataset for normal human was obtained GTEx-TOIL RSEM tpm and screened bone marrow sequencing samples with a sample size of 70. All data data were converted to TPM data and log2 (TPM + 1) processed. This batch effect was removed in all data set merging processes.

### Gene differential expression analysis

Firstly, gene differential expression analysis was realized using the ‘limma’ package in R Studio. The cut-off criteria were |log2fold-change|> 1 and P < 0.05. Secondly, after the gene differential expression analysis was completed, PPI networks of differential genes were constructed using the STRING protein interactions database (https://cn.string-db.org/), and key genes were screened using the 11 topology methods of the CytoHubba plugin in Cytoscape. Finally, Metascape software (https://metascape.org/) was used for gene function enrichment analysis.

### Fuzzy C-Means clustering

The ‘Mfuzzy’ package [8] in R Studio was originally developed as a clustering method for processing gene expression or protein expression profile data, with the core algorithm based on Fuzzy C-Means Clustering (FCM). This method is used to cluster the key genes screened by CytoHubba to further determine the final features.

### XGboost

eXtreme Gradient Boosting (XGBoost), is a Boosting algorithm that implements machine learning algorithms in the framework of Gradient Boosting. XGBoost provides Parallel Tree Boosting (also known as GBDT, GBM) to solve regression or classification problems quickly and accurately. In this study, after the fuzzy C-mean clustering analysis, XGboost regression models were constructed separately for each cluster and the optimal parameters were determined using fivefold cross-validation and grid search method.

### Recursive feature elimination based on cross validation

Recursive feature elimination is essentially a greedy algorithm for finding the optimal subset. The main idea is to repeatedly construct the model and then select the best (or worst) features, select the selected features, and then repeat the process on the remaining features until all the features have been traversed. The order in which the features are eliminated in this process is the ranking of the features. Since purely recursive feature elimination is prone to overfitting problems, cross validation based recursive feature elimination has emerged. In this study, recursive feature elimination based on fivefold cross-validation is used to filter the variables.

### Random ferns classifier

Random Ferns is a classifier based on the plain Bayesian algorithm and is often viewed as a non-hierarchical random forest [9]. The process of constructing the ‘fern’ differs from the decentralized structure of the ‘tree’ constructed by the random forest, but a vertical structure. In addition, the key parameter ‘depth’ must be in the range [1, 16]. In order to find the optimal parameters, tenfold cross-validation repeated three times and grid search method were applied to determine the optimal parameters. In this study, a random fern classification model was constructed to identify bone marrow diagnostic biomarkers for AML.

### Dimensionality reduction algorithm

t-SNE, UMAP, and PCA are three different dimensionality reduction algorithms. Dimensionality reduction algorithms actually recalculate two or three new variables for a number of variables in the sample to achieve the purpose of extracting and synthesizing valid information and rejecting useless information. In other words, the generation of new variables after dimensionality reduction is based on all the previous variables, and together with the grouping information of the sample, the purpose of checking whether the grouping is correct or not can be achieved.

### Online analysis platform

Drug sensitivity analysis was performed by CTD database (http://ctdbase.org) and CTRP database based on GSCA platform (http://bioinfo.life.hust.edu.cn/GSCA/#/). SNV analysis was performed based on GSCA platform. Validation of gene expression was performed based on the GEPIA2 platform (http://gepia2.cancer-pku.cn/#index) and BioGPS database (biogps.org/). ceRNA network construction was performed based on data from the ENCORI database (http://starbase.sysu.edu.cn/), miRWalk database (http://mirwalk.umm.uni-heidelberg.de/), and HMDD database (http://www.cuilab.cn/hmdd/). Survival and prognostic analysis based on Kaplan Meier Plotter database (http://kmplot.com/analysis/index.php?p=background).

## Results

### Gene differential expression analysis

**Fig. 1:**
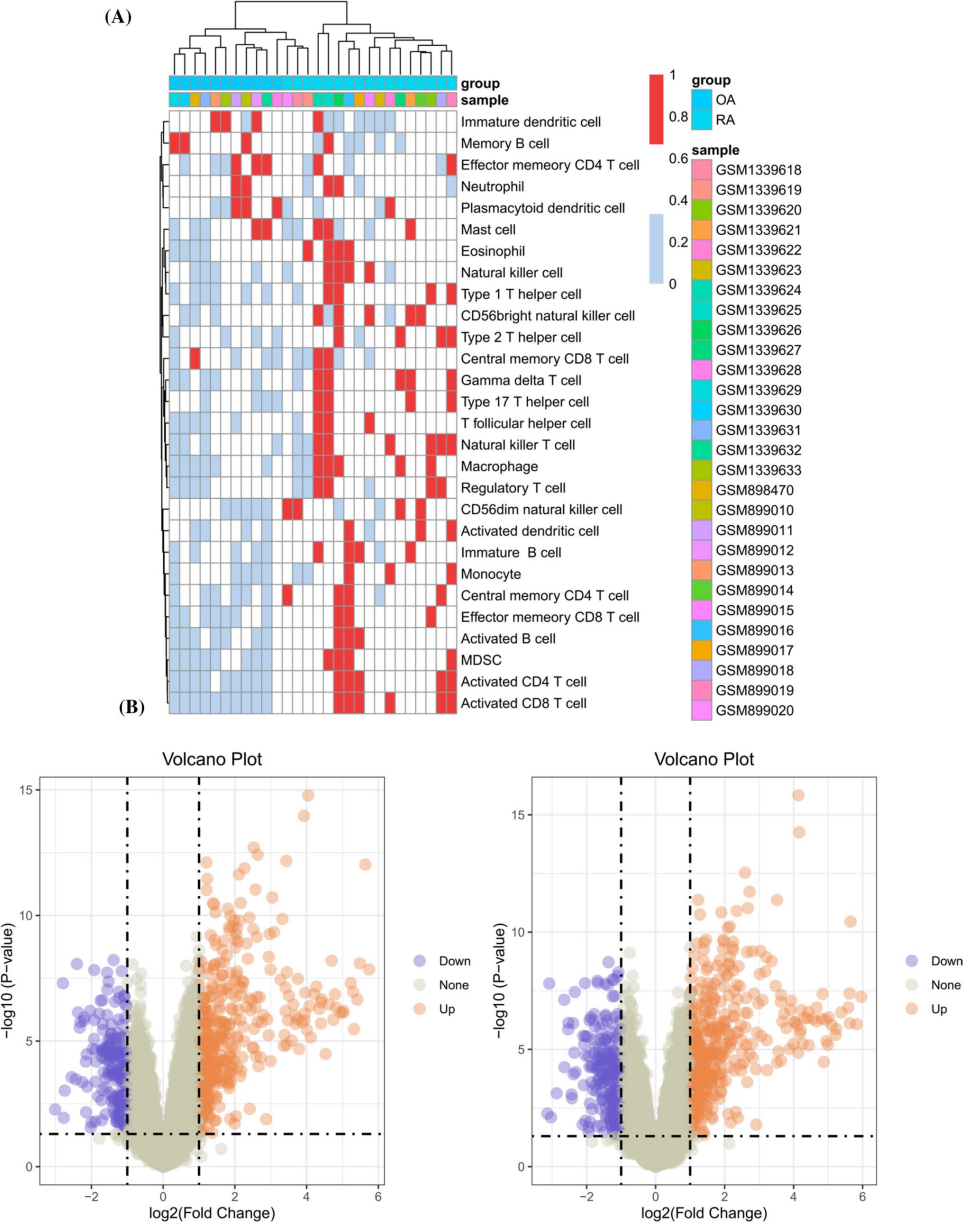
Results of two gene differential expression analyses (**A**) Immune infiltration status of all samples (**B**) Number of differential genes up-regulated and down-regulated

**Fig. 2:**
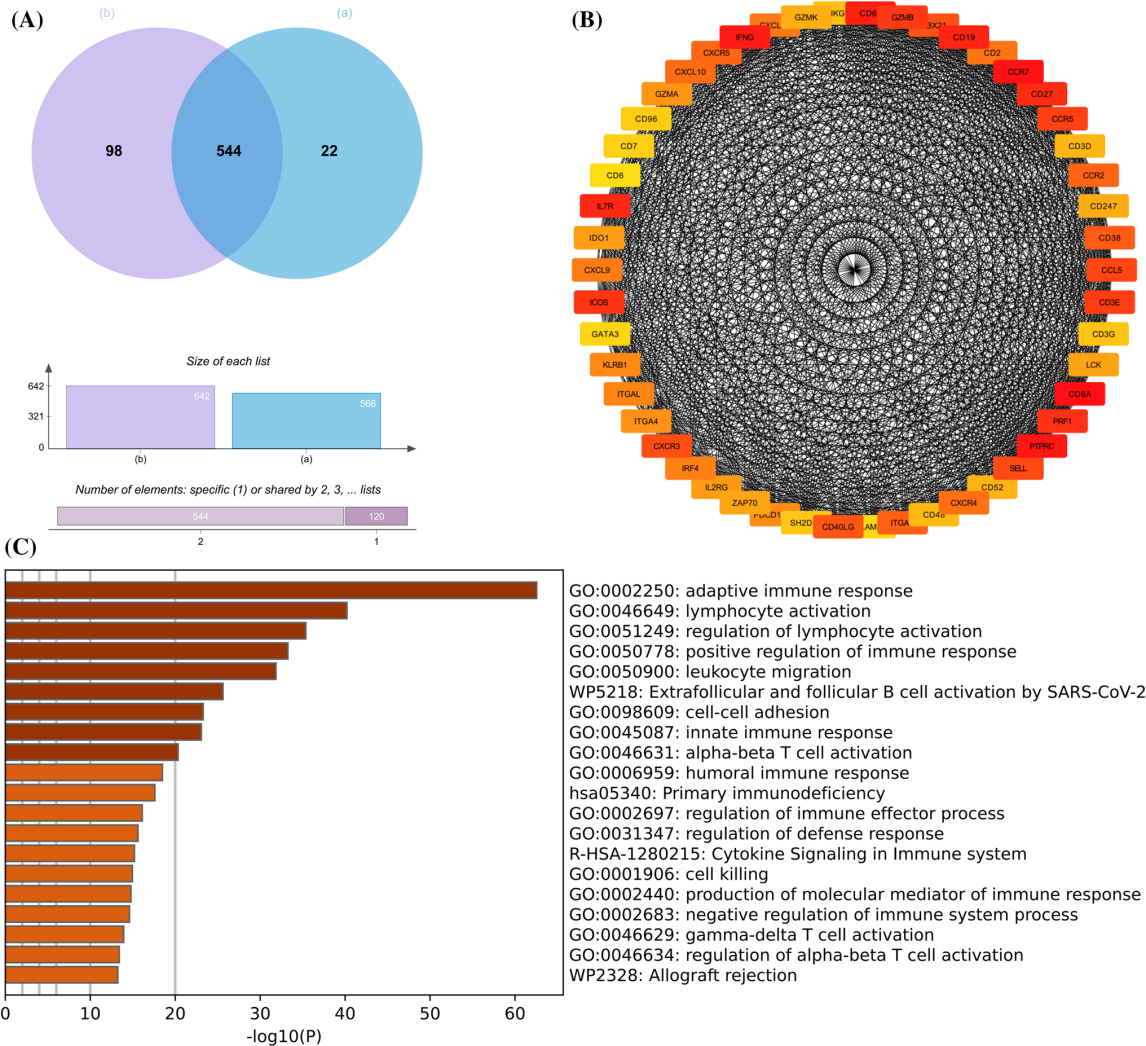
Functional and pathway enrichment analysis of differentially expressed genes in RA and OA (**A**) Number of overlapping differentially expressed genes in RA and OA before and after immune infiltration assessment (**B**) Protein Interaction Network Screening for pivotal genes (**C**) Functional and pathway enrichment analysis of pivotal genes

We evaluated the immune infiltration status of each sample using ssGSEA (Fig. 1A). In order to reduce research error, we excluded three OA samples, so that the group with high immune infiltration scores and the group with low scores were divided into the RA group and the OA group, respectively. Gene differential expression analysis was conducted on the above groups, resulting in 566 differentially expressed genes. Differential gene expression analysis was performed on RA and OA groups without ssGSEA, and 642 differentially expressed genes (DEGs) were identified (Fig. 1B). Take the intersection of the two parts to obtain 544 DEGs (Fig. 2A), and then use the STRING database and the CytoHubba plugin in Cytoscape to obtain 50 hub genes (Fig. 2B). Functional and pathway enrichment analysis of these differential genes showed that most of these genes were enriched in adaptive immune response, lymphocyte activation, positive regulation of immune response, primary immune response, lymphocyte activation (Fig. 2C).

### Screening of pivotal genes

**Fig. 3:**
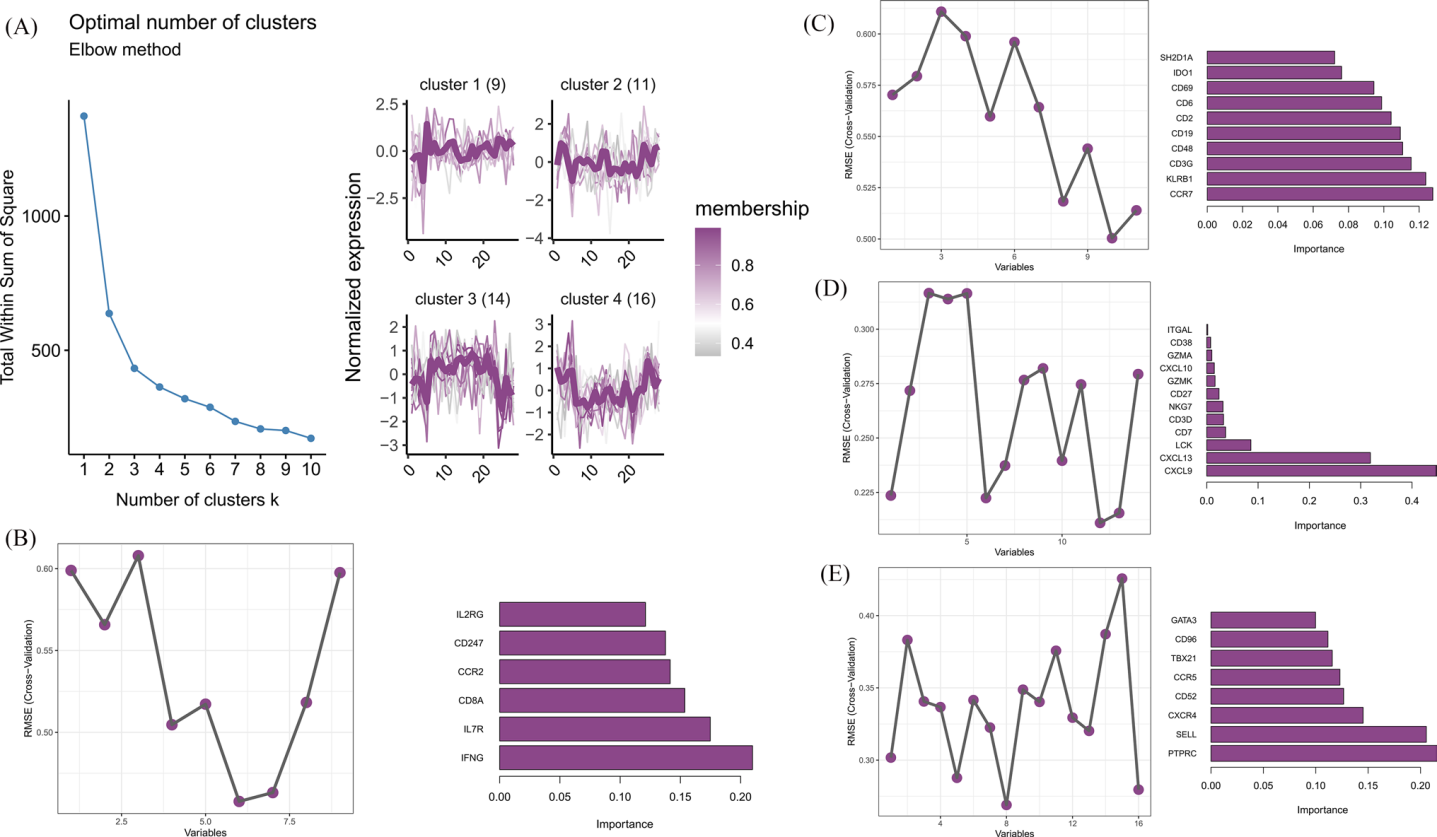
Identify key genes (**a**) Fuzzy C-means clustering results (**b**–**e**) The importance of genes in each cluster

Fuzzy C-means clustering analysis was performed on these 50 pivotal genes, and the results showed that these 50 pivotal genes were clustered into 4 classes (Fig. 3A). The XGboost regression model was constructed separately for each cluster, and the optimal model parameters were determined using fivefold cross-validation and grid search method. The results of the optimal parameters are shown in the table below (Table 1). Then recursive feature elimination based on fivefold cross validation was used to filter the variables, and the results of the number of variables selected according to recursive feature elimination for each model are shown. The first two important variables of each model were selected according to the ranking of importance, and the final identified key genes for RA immune heterogeneity were IFNG, IL7R, CCR7, KLRB1, CXCL9, CXCL13, SELL, and PTPRC (Fig. 3B–E).

**Table 1:** Parameters of each XGboost regression model

	Cluster1	Cluster2	Cluster3	Cluster4
nrounds	50	50	50	100
Max_depth	3	1	1	2
Eta	0.3	0.4	0.3	0.4
Gamma	0	0	0	0
Colsample_bytree	0.8	0.6	0.6	0.6
Min_child_weight	1	1	1	1
Subsample	0.5	0.5	0.5	1

### Immune heterogeneity characterizing RA in AML

**Fig. 4:**
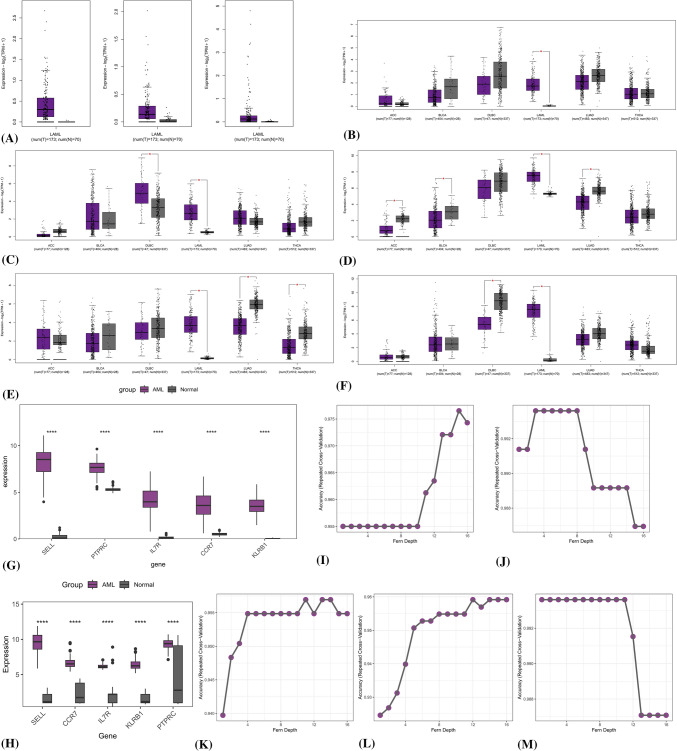
Determination and validation of gene expression and search for the optimal parameters of RFs models (**A**) Expression of IFNG, CXCL9, and CXCL13 in AML bone marrow (**B**–**F**) Expression of IL7R, CCR7, KLRB1, SELL, PTPRC in AML bone marrow and other tumors (**G**) Validation of IL7R, CCR7, KLRB1, SELL, PTPRC expression (**H**) Validation of expression of SELL, PTPRC, IL7R, CCR7, and KLRB1 using BioGPS database (**I**–**M**) Determine the optimal parameters for RFs classification models

Use the 8 genes identified above as the immune heterogeneity characteristic gene set of RA to study their expression levels in AML. The analysis of data in GEPIA2 showed that there was no statistically significant difference in the expression of CXCL9, CXCL13, and IFNG in AML bone marrow (Fig. 4A). There is a statistically significant difference in the expression of IL7R, CCR7, KLRB1, SELL, and PTPRC in AML bone marrow and normal human bone marrow, and the lowest expression value in AML is almost higher than the highest value in the normal group, and this expression difference is more pronounced in AML than in other tumors (Fig. 4B–F). To validate this result, we chose another bone marrow RNA sequencing dataset of AML (n = 151) to merge with the normal samples and remove the batch effect. The results were confirmed by a wilcoxon test of the expression of the two groups, which showed that the differences in the expression of IL7R, CCR7, KLRB1, SELL, and PTPRC were statistically significant between the AML group and the normal group (Fig. 4G). In addition, we conducted another validation of the expression of the above five genes by the BioGPS database (GEO-based cohort data). This validation data included 29 normal and 99 AML cohorts, and the validation results were consistent with our previously obtained results (Fig. 4H).

### Machine learning to identify AML patients

**Fig. 5:**
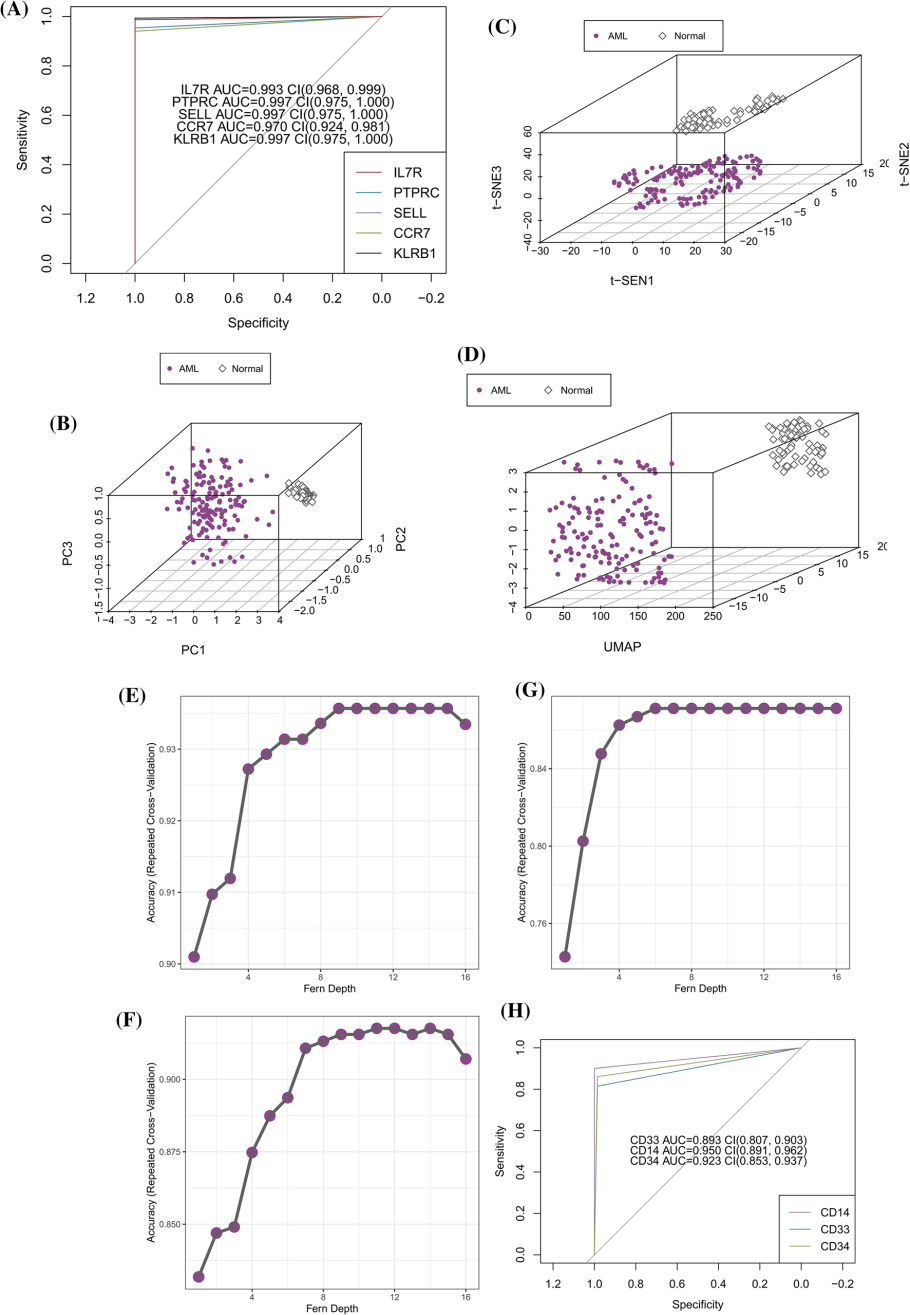
Diagnostic value of bone marrow biomarkers in AML (**A**) ROC analysis results of bone marrow biomarkers in AML (**B**–**D**) UMAP, PCA, t-SNE analysis results (**E**–**G**) Selection of the optimal parameter ‘depth’ for random fern model (**H**) ROC analysis results of traditional AML biomarkers

To identify bone marrow diagnostic biomarkers for AML, a random fern classifier was constructed to distinguish AML patients from normal individuals. The process of constructing the random fern classification model uses tenfold cross-validation repeated three times and grid search method to determine the optimal parameters (Fig. 4I–M). The merged dataset from the previous step was split 70% as the training set and the whole dataset was used as the validation set. The results showed that the AUC value of each biomarker in the validation set was above 0.970 (Fig. 5 A). In addition, the dimensionality reduction algorithm was also used to check whether the grouping was correct. Three algorithms, t-SNE, UMPA, and PCA, were used to analyze the grouping of AML and normal individuals based on these five features and the grouping information of each sample. The results showed that regardless of which algorithm the AML group and the normal group were clearly distinguished (Fig. 5B–D), which further illustrates the value of these five features in diagnosing AML.

In addition, a comparison between old and new AML bone marrow biomarkers was performed.CD33, CD34, and CD14 are AML diagnostic biomarkers that have long been proven by research. Following the same research methodology described above, the optimal parameters of the randomized fern model are shown below (Fig. 5E–G), and the calculated AUC values for CD33, CD34, and CD14 were 0.893, 0.923, and 0.950, respectively (Fig. 5H).

### Immune infiltration analysis

**Fig. 6:**
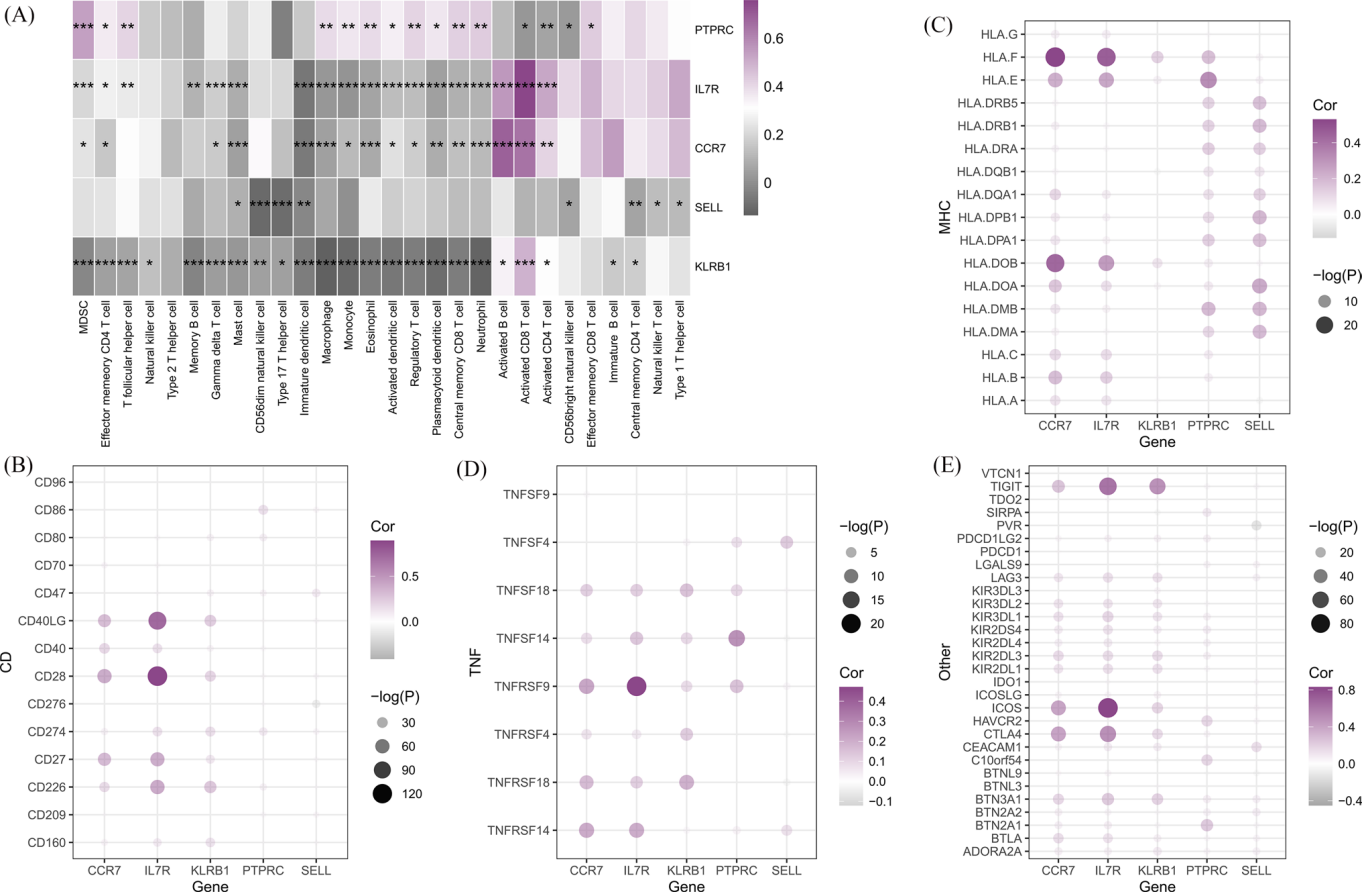
Immune infiltration analysis results (**A**) The correlation between diagnostic genes and immune cells (**B**–**E**) Correlation between diagnostic genes and immune checkpoints

The results indicate that PTPRC is positively correlated with MDSC, and negatively correlated with Activated CD4 T cells and CD56bright natural killer cells. IL7R showed a positive correlation with Activated CD8 T cell, Activated B cell, and Activated CD4 T cell, while a negative correlation with Immature dendritic cell, Macrophage, Monocell, Eosinophel, and Memory B cell. The relationship between the expression of CCR7 and immune cells is similar to that of IL7R, while the expression of SELL shows a negative correlation with all immune cells. The expression of KLRB1 is positively correlated with Activated CD8 T cells and negatively correlated with all other immune cells (Fig. 6A). The expression of these five biomarkers was positively correlated with the expression of common immune checkpoints. In comparison, the expression of SELL is negatively correlated with the expression of more immune checkpoints. PTPRC did not show a particularly significant correlation with the expression of CD family genes, while KLRB1 did not show a particularly significant correlation with the expression of MHC family genes (Fig. 6B–E).

### Drug sensitivity analysis and SNV analysis

**Fig. 7:**
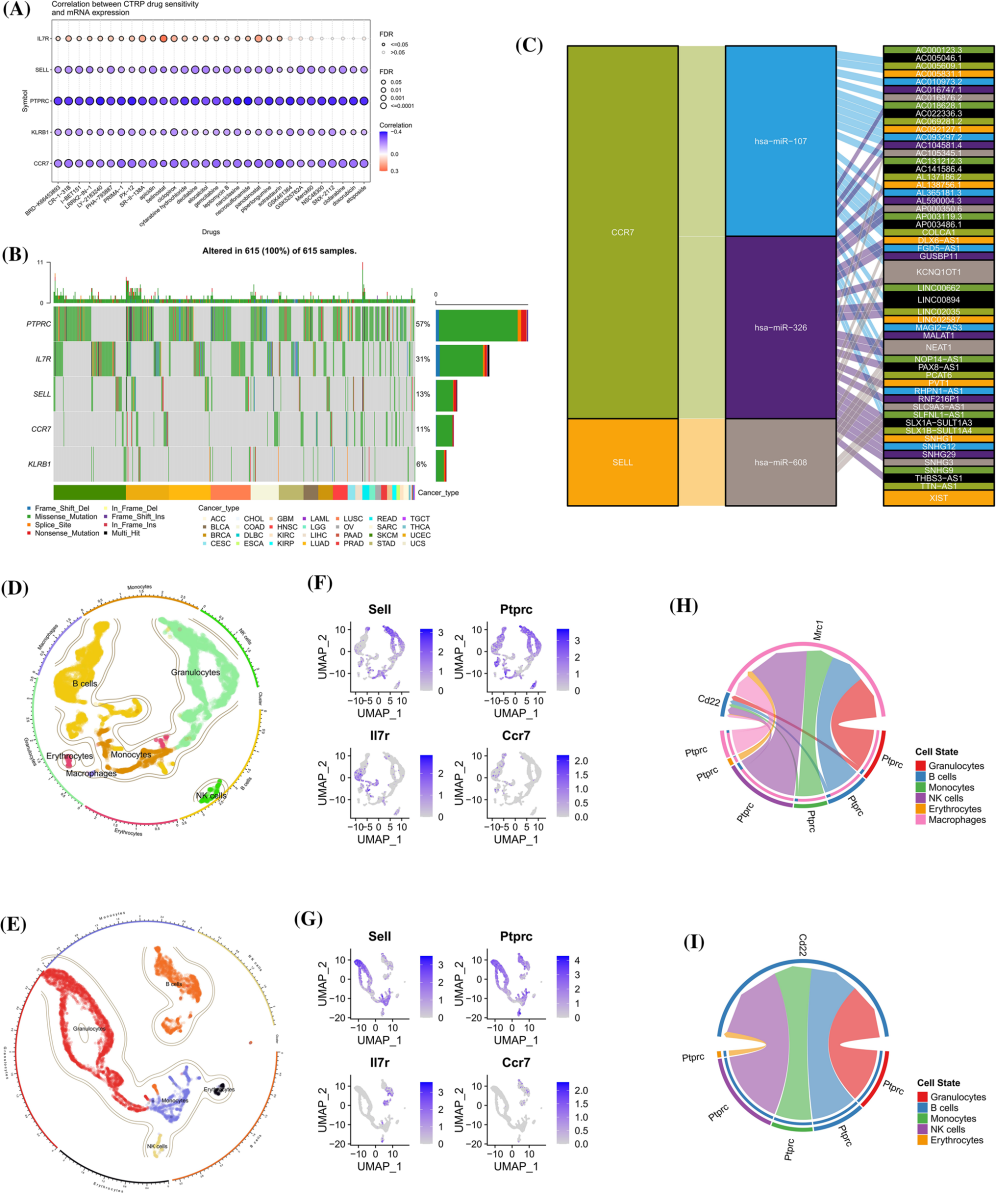
GSCA online analysis and single cell sequencing analysis results (**A**) Drug sensitivity analysis results (**B**) SNV analysis results (**C**) Building a ceRNA network (**D**) Single cell sequencing analysis results in the control group (**E**) Single cell sequencing analysis results of RA group (**F**) Expression distribution of target genes in various cells of the control group (**G**) Expression distribution of target genes in various cells of the RA group (**H**) CD45 signaling pathway in the control group (**I**) CD45 signaling pathway in the RA group

The relationship between the expression of IL7R, CCR7, KLRB1, SELL, PTPRC and drug sensitivity was analyzed by CTRP database. The results showed that the expression of IL7R showed a negative correlation with the sensitivity of apicidin, belinosta, ciclopirox, cytarabine hydrochloride, and panobinostat, and that IL7R may affect the therapeutic efficacy of AML patients (Fig. 7A). A search for drugs that could reduce IL7R expression in the CTD database showed that acetylcysteine was an effective drug for reducing IL7R expression. Missense mutations were the most common in pan-cancer SNV analysis of target genes, and PTPRC had the highest proportion of SNVs in pan-cancer samples, which was particularly prominent in small cell lung cancer (Fig. 7B).

### Construction of ceRNA network

1386 miRNAs related to diagnostic genes were obtained from the miRWalk database, and 178 miRNAs related to AML were obtained from the HMDD database. 2 mRNAs were obtained by taking their intersection: SELL and CCR7, and 4 miRNAs: hsa-miR-107, hsa-miR-1299, hsa-miR- 608, hsa-miR-326. 3 miRNAs and 54 lncRNAs were obtained by searching for miRNA-lncRNA relationship pairs from ENCORI database for these 4 miRNAs then the ceRNA network composed of mRNA-miRNA-lncRNA was constructed based on the above results. The results are shown below (Fig. 7C).

### Analysis of single cell sequencing data for RA and control

Analysis of bone marrow single-cell sequencing data from RA and control mice showed that the expression of SELL and PTPRC was significantly stronger in the RA group than in the control group (Fig. 7D–G). Important interaction pairs associated with the CD45 signaling pathway in the RA group were exclusively contributed by Ptprc-Cd22, whereas important interaction pairs associated with the CD45 signaling pathway in the control group were mainly contributed by Ptprc-Mrc1, with Ptprc-Cd22 contributing only a very small fraction (Fig. 7H, I).

### The correlation between the expression of immune heterogeneity characteristic genes and different subtypes of AML

**Fig. 8:**
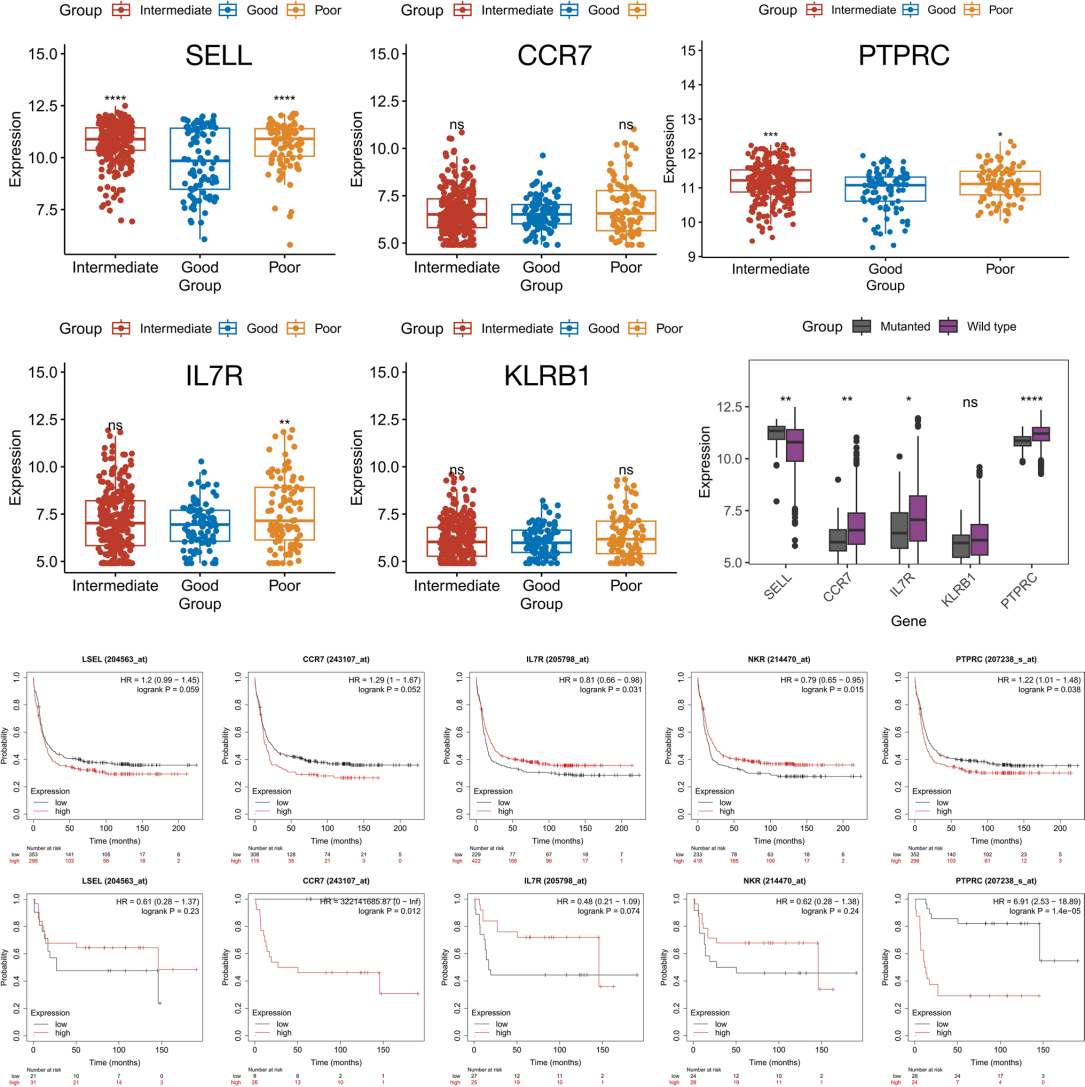
The relationship between different gene expressions and AML subtypes (**A–E**) Differential expression of different genes under different cytogenetic risk groupings (**F**) Differential expression of different genes under different CEBPA mutation types (**G**) Survival and prognosis analysis of patients with wild-type mutations under different gene expression patterns (**H**) Survival and prognosis analysis of patients with mutanted mutations in different gene expression patterns

We first analyzed the expression of SELL, CCR7, IL7R, KLRB1, and PTPRC in relation to different cytogenetic risks in dataset GSE6891. We found that the expression of SELL, IL7R, and PTPRC was significantly higher in patients with cytogenetic risk of poor than in patients with cytogenetic risk of good (Fig. 8A–E). We divided AML patients into two groups of high and low expression according to the median of their respective expression of these five genes, and then analyzed the relationship between different expression status of different genes and different cytogenetic risks, we found that only the expression of SELL was associated with different cytogenetic risks (r = 0.104, *P* < 0.05) (Table 2). Then we further investigated the association of SELL, CCR7, IL7R, KLRB1, PTPRC with different CEBPA mutation types. The results showed that for different mutation types, the expression of the above five genes was significantly different (Fig. 8F). According to the same method described above, AML patients were categorized into high and low expression groups, and the expression of all genes except KLRB1 was correlated with different types of CEBPA mutation, and the expression of SELL and PTPRC was more correlated with different types of CEBPA mutation compared with the other three genes (Table 2). Finally, we analyzed the relationship between high and low expression of the above five genes and the prognosis of AML patients according to different CEBPA mutation types. The results showed that the survival probability of patients with high expression of IL7R and KLRB1 was significantly higher in patients with wild-type mutations, which may be associated with a favorable prognosis of the patients, and that the survival probability of patients with high expression of PTPRC was significantly lower, which may be associated with a poor prognosis of the patients (Fig. 8G). In patients with mutation type mutant, high expression of PTPRC also predicted a significantly lower probability of survival, which may also indicate an association with a poor prognosis for the patient (Fig. 8H).

**Table 2:** The relationship between the expression patterns of different genes and the cytological genetic risk and different CEBPA mutation states in AML patients

	Cytogenetic Risk		CEBPA mutation	
	Cor (Spearman)	*P*	Cor (Cramer’s V)	*P*
SELL	0.104	< 0.05	0.152	< 0.05
CCR7	0.014	> 0.05	0.100	< 0.05
IL7R	0.028	> 0.05	0.100	< 0.05
KLRB1	0.035	> 0.05	0.040	> 0.05
PTPRC	0.056	> 0.05	0.238	< 0.05

## Discussion

RA is an autoimmune disease that is associated with an increased risk of not only lymphoproliferative disorders, but also myeloid malignancies, increasing the risk of developing AML [10, 11]. As these studies continue to grow and intensify, this seems to open up the possibility of exploring diagnostic biomarkers from one disease to another. Due to the chronic immune stimulation or bone marrow infiltration of RA, some risk features at the molecular level are progressively manifested before it causes AML, and these risk features are not found in normal individuals, and these molecular features underpinning the development of AML remain largely speculative. Some of the differences between RA and normal individuals at the molecular level are reflected in the immune heterogeneity of RA. Therefore, we will first investigate the immunological heterogeneity features of RA and then use this as a starting point to explore whether these features are relevant to the diagnosis of AML.

Firstly, in this study, we performed two differential expression analyses on RA and OA samples before and after calculating the immune infiltration scores of each sample, and 544 DEGs were obtained by intersecting the results of the two differential analyses. 544 DEGs were obtained through functional and pathway enrichment analyses of these DEGs, and the results showed that these DEGs were mainly enriched in the adaptive immune response, positive regulation of immune response, primary immunodeficiency, leukocyte migration, cytokine signaling in the immune system, and the expression of the immune infiltration scores of each sample response, positive regulation of immune response, primary immunodeficiency, leukocyte migration, and cytokine signaling in Immune system. These enrichment entries are from different databases but the results of functional and pathway enrichment are not very different. In addition, it has been shown that the biological processes of the immune system are crucial for the formation of a complex bone marrow micro-environment [12, 13], so that understanding the immunological heterogeneity of RA will help to develop effective bone marrow markers for AML diagnosis.

Secondly, eight genes associated with RA immune heterogeneity were obtained by fuzzy C-mean clustering and post-tuning XGboost regression model calculations: IFNG, IL7R, CCR7, KLRB1, CXCL9, CXCL13, SELL, and PTPRC. With these eight immune heterogeneity genes of RA as a set of characterized genes to be investigated in AML, we evaluated their expression in the expression in AML. Except for IFNG, CXCL9, CXCL13, which were not statistically significant in AML above normal, the difference between AML and normal was statistically significant for the remaining five genes, and this difference was highly significant. We also validated the expression of these 5 genes using a new dataset and analyzed their diagnostic value for AML using a random ferns classifier. Typically, shed forms of SELL are often studied in conjunction with AML, and plasma from AML patients shows high levels of shed forms of selections [14–16]. Little attention has been paid to the amount of SELL expressed in AML bone marrow. In this study, after analyzing a large amount of data, we found that the expression of SELL is much higher in AML bone marrow than in normal subjects, which provides a new biomarker for bone marrow diagnosis of AML. In addition, it has been found that interferon α up-regulates the expression of SELL [17], and interferon α is often used in the treatment of RA [18, 19], which may bring a potential risk of AML development in RA patients. In this study, KLRB1 and CCR7 were found to be barely expressed in normal human bone marrow, but highly expressed in AML bone marrow, and besides AML, they were also over-expressed in a few types of cancers, such as testicular germ cell tumors (TGCT), kidney renal clear cell carcinoma (KIRC), pancreatic cancer (PAAD), etc., and the tissues of the three cancers studied were not closely related to the bone marrow, so KLRB1 and CCR7 will be bone marrow biomarkers helpful for the diagnosis of AML. Previous studies have shown that over-expression of PTPRC can predict poor prognosis and serve as a therapeutic target for pediatric AML [20]. In the present study PTPRC can be used as a biomarker for AML diagnosis. Earlier studies have shown that IL7R is extremely low in bone marrow [21], which corroborates with this paper, so the presence of a significant increase in bone marrow of AML patients combined with the results of ROC analysis (AUC = 0.993) suggests that IL7R is a suitable bone marrow biomarker for AML diagnosis.

And then, this study analyzed the relationship between these five genes and the AML immune microenvironment and AML therapeutic drug sensitivity, and attempted to construct a ceRNA network based on these five genes. This study found that KLRB1 can be a target for AML therapy. In a previous study on glioma, it was shown that activation of CD161 would weaken T-cell responses to tumor cells, thereby inhibiting the utility of immunotherapy. Knockdown of KLRB1, the gene encoding CD161, strongly enhanced the ability of T cells to attack glioma cells and reduced T cell exhaustion [22]. In this study, KLRB1 showed almost negative correlation with different types of T cells and positive correlation with almost all common immune checkpoints, and its expression level was also much higher than that of normal individuals. Therefore, this study suggests that KLRB1 could be a new target for AML treatment or could be used in the development and design of novel immunotherapeutic drugs. This study also found that IL7R affects chemotherapy resistance. In fact, some studies have already confirmed that IL7R is related to chemotherapy resistance [23–26], so this study found a drug that can reduce the expression of IL7R through the Drug Interaction Database: acetylcysteine, which may be helpful in reducing chemotherapy resistance. To fully understand the role of these diagnostic genes in AML, this study also constructed a ceRNA network. miRNAs and lncRNAs play key roles in gene regulation and cancer biology. miRNAs are small non-coding RNAs that are known to bind and control mRNA expression [27–31]. In the ceRNA network, lncRNAs act as miRNAs to regulate gene expression and participate in cancer development. The construction of the ceRNA network will better investigate the role of diagnostic gene genes in AML and their potential mechanisms. In the results of the cell communication analysis of RA and control mice in this study, it is not difficult to see that completely different from the control group, the CD45 signaling pathway in RA is exclusively contributed by Ptprc-Cd22, which in turn is a biomarker for a variety of malignant tumors such as leukemia and non-Hodgkin’s lymphoma, which may suggest that the CD45 signaling pathway is of great importance to be investigated in the transition from RA to leukemia.

Finally, we analyzed the relationship between the expression of these five biomarkers and the different cytogenetic risks of AML patients with different CEBPA mutation status. We found that the expression of SELL was higher the worse the cytogenetic risk of AML, while the expression of PTPRC was highest in the group with Intermediate cytogenetic risk. The expression of SELL was significantly higher in patients with CEBPA mutation type as mutanted than in wild type patients. The expression of all genes except SELL was significantly higher in patients with wild type than in patients with mutated type. In addition, different expression patterns of biomarkers other than KLRBA correlated with CEBPA mutated type, although this correlation was not strong. The above results demonstrate that the expression of genes characterizing the immune heterogeneity associated with RA is associated with different cytogenetic risks and CEBPA mutation types in AML.

This study explored the relationship between genes characterizing the immune heterogeneity of RA and AML, but further research is needed for an in-depth exploration of the transition from RA to AML. Indeed, the low frequency of RA developing into AML may in some cases be associated with certain somatic mutations, such as UBA1 in Vexas syndrome. One study identified Vexas syndrome as a myeloid-driven inflammatory disorder caused by somatic mutations in the UBA1 gene, further exposing the increasingly recognized overlap between hematologic disorders and autoimmune and/or autoinflammatory manifestations [32]. It has also been demonstrated that there is a commonality in the mechanistic pathways involved in RA, AML, and Vexas syndrome [33], and that all involve a profound alteration of the inflammatory response and the bone marrow microenvironment. Somatic mutations in the ASXL1 gene may also be a contributing factor in the progression of RA to AML. Studies have shown that somatic mutations in the ASXL1 gene correlate with RA [34], and ASXL1 mutations are highly specific in diagnosing AML [35]. In addition, mutations in DNMT3A and TET2, which are closely associated with RA, may also be important in the progression of RA to AML [36, 37].

Although this study analyzed a large amount of data using machine learning and bioinformatics methods to identify five biomarkers associated with AML, there are certain shortcomings. One is that the study may have been affected by potential confounding factors. The first is that although OA was chosen as a control in this study to reflect the immune heterogeneity of RA, after all, the mechanistic pathways of RA and OA are different, and to some extent this may interfere with the data analysis. Secondly, it is not clear whether the RA or OA samples had received treatment, as some drugs may have affected their immune function during the treatment process, which may have resulted in altered expression of certain genes. Despite some confounding, this study explored the relationship between genes that characterize immune heterogeneity in RA and AML, demonstrating that these genes are useful in distinguishing healthy cells from leukemic cells.

## Conclusion

Current research evidence clearly demonstrates the association of SELL, PTPRC, IL7R, CCR7, and KLRB1 with AML, which are critical for distinguishing healthy cells from leukemic cells in AML. In addition, KLRB1 is expected to be a new target for AML treatment.

## Data Availability

In this study data are available from the GEO database (http://www.ncbi/nlm.nih.gov) and ucsc xena database (https://xenabrowser.net/datapages/) to download.
